# Case report: Efficacy analysis of radiofrequency catheter ablation combined with atrial appendage resection for atrial tachycardia originating from the atrial appendage in children

**DOI:** 10.3389/fcvm.2022.990325

**Published:** 2022-10-18

**Authors:** Jing Liu, Xiaoxiao Cao, Changjian Li, Xiaoyuan Feng, Dongming Sun, Yong Zhang

**Affiliations:** ^1^Department of Cardiology, Wuhan Children’s Hospital (Wuhan Maternal and Child Healthcare Hospital), Tongji Medical College, Huazhong University of Science and Technology, Wuhan, China; ^2^Department of Cardiac Ultrasound, Wuhan Children’s Hospital (Wuhan Maternal and Child Healthcare Hospital), Tongji Medical College, Huazhong University of Science and Technology, Wuhan, China

**Keywords:** case report, heart surgery, cardiology, atrial tachycardia, atrial appendage, tachycardia-induced cardiomyopathy, radiofrequency catheter ablation, atrial appendage resection

## Abstract

**Objective:**

The aim of this study was to investigate the efficacy of radiofrequency catheter ablation (RFCA) combined with atrial appendage (AA) resection to treat atrial tachycardia (AT) originating from the AA in children.

**Materials and methods:**

Using the Ensite three-dimensional electroanatomic mapping system, three children with AT originating from the AA were diagnosed. Clinical features and electrocardiographic (ECG) manifestations were analyzed. Ablations were performed using a cold saline-infused catheter at appendages targeting loci of AT origin under the guidance of the Ensite system. Atrial appendage resection was performed in combination with cardiac surgery, and the curative effect was evaluated.

**Results:**

The ages of the three patients were 3.5, 5.75, and 12.9 years. Two cases originated from the right atrial appendage (RAA) and one originated from the left atrial appendage (LAA). The ECG characteristics of AT from the RAA were as follows: (1) negative P waves in lead V1; (2) positive P waves in leads II, III, and aVF; (3) positive P wave in lead I with varying shapes in lead aVL; and (4) prolonged PR interval with no QRS wave after some P waves. The ECG of the LAA was characterized by (1) positive P waves in lead V1 with a bimodal pattern; (2) positive P waves in leads II, III, and aVF; and (3) negative P waves in leads I and aVL. Preoperative echocardiography showed cardiac enlargement and a decreased left ventricular ejection fraction (LVEF) in all three cases. One case was cured after RFCA, and the remaining two cases required AA resection after RFCA. No recurrence was detected at 1–18 months of follow-up, and the left ventricular end-diastolic diameter and LVEF returned to normal.

**Conclusion:**

Atrial tachycardia originating from the AA in children showed a characteristic P-wave presentation on ECG, and sustained episodes of AT resulted in tachycardia-induced cardiomyopathy. Children who are not successfully controlled by RFCA or who have a recurrence after RFCA could benefit from AA resection.

## Introduction

Atrial tachycardia (AT) originating from the atrial appendage (AA) is clinically characterized by palpitation, chest distress, dyspnea, and other non-specific manifestations. Persistent AT is the electrocardiographic (ECG) characteristic of AT originating from the AA, and approximately 25–50% develops tachycardia-induced cardiomyopathy (TIC), which is characterized by increased left ventricular end-diastolic diameter (LVEDD) and decreased left ventricular ejection fraction (LVEF) (1, 2). Atrial tachycardia originating from the AA in children accounts for approximately 30–50% of AT cases (3), which is higher than that in adults. In a study of 60 children with AT by Gulhan et al., AT originating from the left atrial appendage (LAA) and right atrial appendage (RAA) accounted for 15.8 and 15.5% of cases, respectively (4).

Antiarrhythmic drugs do not adequately mitigate AT, and catheter radiofrequency ablation (RFCA) is often used to treat AT. The success rate of RFCA is variable. Freixa (5) reported that 15 patients with RAA tachycardia had no recurrence after RFCA, whereas Yamada (6) reported that 13 patients with LAA tachycardia had no recurrence after RFCA. The recurrence of AT following RFCA maybe 10–20% (7). In cases of recurrence, a second ablation or other treatments were needed (8). For cases of ablation failure or recurrence after ablation, AA resection was an option (9).

In this report, three children were diagnosed with AT using surface ECG and were determined to have originated from the AA using Ensite’s three-dimensional mapping system. Each of the patients was treated using RFCA. Notably, one patient was successfully ablated immediately, one patient experienced ablation failure, and one patient underwent successful ablation but experienced recurrence. In the latter two cases, small incision LAA and thoracoscopic RAA resection were performed during cardiothoracic surgery.

## Subjects and materials and methods

### General information

In this study, three children with AT who were admitted to the Department of Cardiovascular Medicine, Wuhan Children’s Hospital for RFCA to treat AT that originated from the AA were included. The ages were 3.5, 5.75, and 12.9 years, with two girls and one boy. Antiarrhythmic drugs failed to convert the patients to sinus rhythm. Atrial tachycardia was determined to originate from the LAA in one case and from the RAA in two cases, as determined by intracardiac mapping and RFCA.

### Diagnostic criteria

Atrial tachycardia was diagnosed based on the following surface ECG criteria (10): (1) narrow QRS tachycardia and (2) P waves present in the front of the QRS waves or hidden in the QRS or T waves, resulting in differences from sinus P shape.

### Surgical protocol

#### Electrophysiological examination

Before surgical procedures, children stopped using antiarrhythmic drugs for more than 5 half-lives. During the operation, 100°U/kg of intravenous heparin was given for anticoagulation. Aspirin (3–5 mg/kg/day) was administered orally for 1°month after any operation involving the LAA. The electrophysiological examination was performed under general anesthesia without intubation. After the puncture through the left subclavian vein, a 6F vascular sheath was inserted, and the coronary sinus mapping electrode was inserted. After the puncture through the left femoral vein, a 6F vascular sheath was placed near the right ventricular electrode. The ablation catheter was delivered after inserting an 8F vascular sheath through the right femoral vein puncture.

#### Radiofrequency ablation

(1) Using the Ensite system, atrial modeling and AT activation mapping were performed, and three-dimensional images of the anatomy and activation sequence of the atrium and AA were constructed. If AT originated from the left atrium, a puncture was performed through the atrial septum, and the ablation catheter was introduced into the left atrium through the long sheath for mapping. Each part of the cardiac chamber was marked using different colors based on excitation times. Red was the earliest excitation point, and purple was the last. The earliest excitation point was identified through accurate mapping of the red area. If the rough mark showed the origin of the deep part of the AA or the special shape of the AA, it was necessary to perform radiography first, and then perform fine mapping after clearly showing the shape of the AA.

(2) A cold saline infusion catheter or a pressure cold saline infusion catheter was selected, and 17 ml/min of cold saline was introduced to maintain a temperature of 43°C and a power of 20–35 W. If the ablation was effective, the ablation was consolidated for 60 s (s) per point. AT was not observed 20 min after ablation. Atrial program stimulation after intravenous isoprenaline drip and regular atrial program stimulation did not induce AT.

#### Atrial appendage resection

Atrial tachycardia originating from the AA that was not controlled by RFCA was treated by AA resection as follows. (1) Thoracoscopic RAA resection. Incisions were made among the eighth intercostal space of the right posterior axillary line, the fourth intercostal space of the middle axillary line, and the fifth intercostal space of the anterior axillary line. A thoracoscope, operating forceps, and electrocoagulation hook were placed in the Trocar. The right pericardium was opened to expose the right atrium and RAA. Using non-invasive forceps, the RAA was pulled open and removed using an endoscopic cutting stapler. (2) LAA resection. A posterolateral incision was made in the left chest, and the pericardium was opened to expose the left atrium and LAA. The root of the LAA was clamped with a C-clamp, and the LAA was completely removed. The excised left and right AA was sent for pathological examination.

#### Follow-up

All patients underwent continuous 12-lead ECG, 24-h ambulatory ECG, and echocardiography before discharge. The patients were followed up using 12-lead ECG and echocardiography, and if necessary, a 24-h ambulatory ECG, at 1, 3, 6, and 12 months, and every year thereafter.

## Results

In this study, three cases of AT originating from the AA were included, with a history of 3 days to 2 years of persistent AT that failed to respond to antiarrhythmic drugs. Echocardiography showed an enlarged heart with LVEDDs of 40, 45.8, and 45 mm, and LVEFs of 42, 29, and 45%, respectively (Table 1).

**TABLE 1:** Clinical features and efficacy analysis of three cases of atrial tachycardia originating from the atrial appendage.

		Case 1	Case 2	Case 3
Gender		female	female	male
Age		3.5 years	5.75 years	12.9 years
Body mass (kg)		15	23	34
Chief complaint		Tachycardia was found for 5 days	Syncope once 3 days ago	Palpitation for 2 years
AT frequency (bpm)		200	160	145
CK-MB (U/L)		26	17	17
Troponin T (ng/ml)		0.068	0.113	0.066
BNP (pg/ml)		> 9000	2768\	181
Preoperative ECG		Sustained AT	Sustained AT	Sustained AT
The orgin of AT		RAA	LAA	RAA
Preoperative LVEDD (mm)		40	45.8	45
Preoperative LVEF (%)		42	29	45
antiarrhythmic agents		amiodarone	cedilanid, amiodarone	propafenone, amiodarone, digoxin, betaloc
treatment		RFCA	RFCA combined with LAA resection	RFCA combined with thoracoscopic RAA resection
Postoperation of RFCA	ECG	sinus rhythm	paroxysmal AT	AT
	LVEDD (mm)	39	-	-
	LVEF (%)	50	-	-
Postoperation of AA resection	ECG	-	sinus rhythm	sinus rhythm
	LVEDD (mm)		42.6	44
	LVEF (%)		33	52
1°month after the operation	ECG	sinus rhythm	sinus rhythm	sinus rhythm
	LVEDD (mm)	36	37	42
	LVEF (%)	53	46	56
1°year after the operation	ECG	sinus rhythm	sinus rhythm	-
	LVEDD (mm)	35	35	
	LVEF (%)	65	60	

bpm, beats per minute; CK-MB, creatine kinase-MB; BNP, brain natriuretic peptide; ECG, electrocardiogram; AT, atrial tachycardia; RAA, right atrial appendage; LAA, left atrial appendage; LVEDD, left ventricular end-diastolic diameter; LVEF, left ventricular ejection fraction; RFCA, radiofrequency catheter ablation; AA, atrial appendage.

**FIGURE 1:**
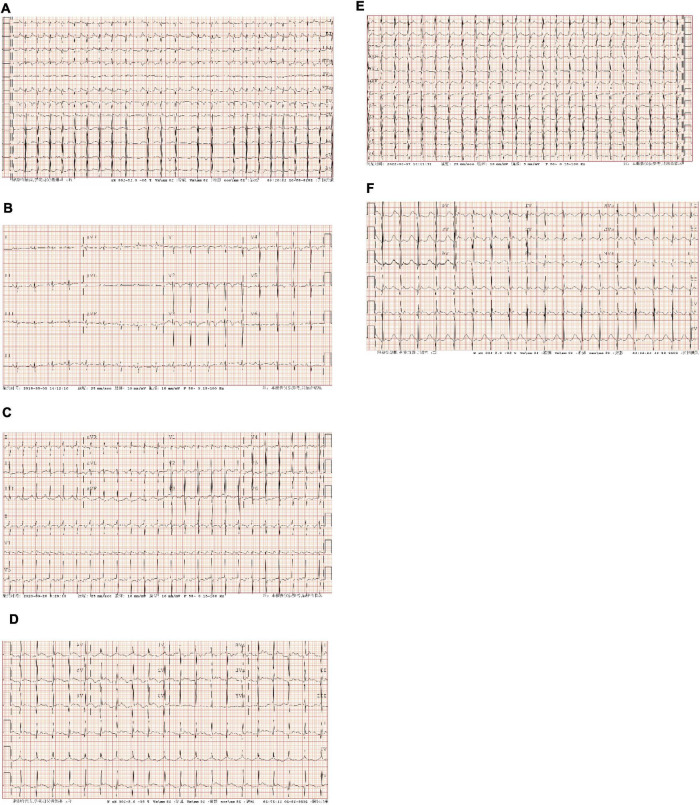
Preoperative and postoperative electrocardiographic (ECG) of atrial tachycardia (AT) from atrial appendage (AA). **(A)** Case 1: persistent AT. The ventricular rate was 137–200 bpm; P waves in lead V1 were negative, and P waves in V3–V6 gradually moved in the positive direction; P waves in leads I, II, III, and aVF were positive, and P waves in lead aVL were negative; the PR interval was prolonged, with no QRS wave after partial P wave. **(B)** Case 1: ECG after radiofrequency catheter ablation (RFCA). Sinus rhythm; P waves in leads I, II, III, aVF, and V1–V6 were positive, and the HR was 110 bpm. **(C)** Case 2: persistent AT. The ventricular rate was 142 bpm; P waves in lead V1 were positive and bimodal; P waves in leads II, III, and aVF were positive; P waves in leads I and aVL were negative. **(D)** Case 2: ECG after left atrial appendage (LAA) resection. sinus rhythm; P waves in leads I and aVL were positive, and the HR was 120 bpm. **(E)** Case 3: persistent AT. The ventricular rate was 145 bpm; P waves in lead V1 were negative, and P waves in leads V2–V6 gradually moved in the positive direction; P waves in leads I, II, III, aVF, and aVL were positive; the PR interval was prolonged. **(F)** Case 3: ECG after RAA resection. Sinus rhythm; P waves in leads I, II, III, aVF, and V1–V6 were positive, and the HR was 108 bpm. AT, atrial tachycardia; bpm, beats per minute; RFCA, radiofrequency catheter ablation; ECG, electrocardiogram; LAA, left atrial appendage; RAA, right atrial appendage; HR, heart rate.

Electrocardiogram characteristics of AT originating from the RAA in 2 cases included the following: (1) P waves in lead V1 were negative, and the precordial lead gradually moved in the positive direction; (2) P waves in leads II, III, and aVF were positive; (3) P waves in lead I were positive; and (4) the PR interval was prolonged, with no QRS waves after some P waves (Figure 1).

Electrocardiogram characteristics of AT originating from the LAA in 1 case were as follows: (1) P waves in lead V1 were positive and double-peaked; (2) P waves in leads II, III, and aVF were positive; and (3) P waves in lead I and aVL were negative (Figure 1).

**FIGURE 2:**
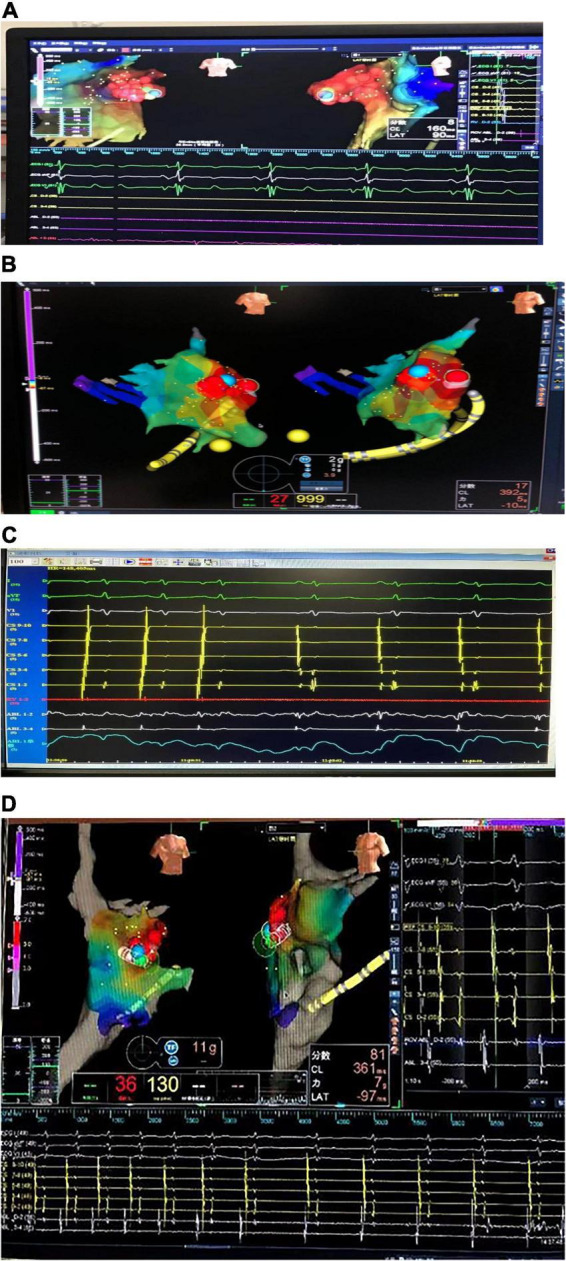
Three-dimensional electroanatomical mapping. **(A)** Three-dimensional electroanatomical mapping of case 1 showed the atrial tachycardia (AT) from right atrial appendage (RAA): the earliest excitation point in the red area is the RAA, the red circle is the ablation target, and the blue circle is the best ablation target. **(B)** Three-dimensional electroanatomical mapping of case 2 showed the AT from left atrial appendage (LAA): the earliest excitation point in the red area is LAA, the red circle is the ablation target, and the blue circle is the best ablation target. **(C)** The electrogram of the target site of case 2: during the ablation, the atrial activation sequence was changed from CS1-2 to CS9-10 to CS9-10 to CS1-2. **(D)** Three-dimensional electroanatomical mapping of case 3 showed the AT from RAA: the earliest excitation point in the red area is the RAA, the red circle is the ablation target, and the blue circle is the best ablation target. The sequence of atrial activation did not change significantly, but the shape of the A wave and the AA interval was changed. AT, atrial tachycardia; LAA, left atrial appendage; RAA, right atrial appendage.

**FIGURE 3:**
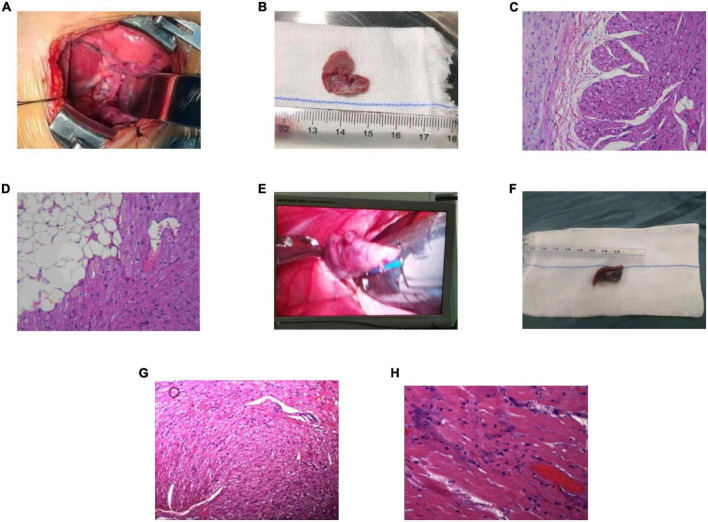
Excised atrial appendage and pathology (200×). **(A)** Case 2: the image of the left atrial appendage (LAA) during the operation. **(B)** Case 2: the excised LAA. **(C,D)** Case 2: postoperative pathology. Striated muscle and fibrous adipose tissue of LAA, local tissue degeneration. **(E)** Case 3: the image of the right atrial appendage (RAA) during the operation. **(F)** Case 3: the excised RAA. **(G,H)** Case 3: postoperative pathology. The arrangement of myocardial fibers in the RAA was disordered, some myocardial nuclei were enlarged, some muscle fibers showed granular degeneration and vacuoles, and tiny necrosis foci could be seen locally. LAA, left atrial appendage; RAA, right atrial appendage.

Three children with AT from the AA were treated by RFCA. In case 1, the earliest excitation point was marked at the peak of RAA during the operation. The catheter was perfused with cold saline, and the ablation parameters (43°, 35 W, 17 ml/min) were selected, and the pressure was controlled at 5–10 g. Atrial tachycardia was terminated after acceleration, and consolidation ablation was performed four times for 60 s. Atrial tachycardia could not be induced by regular atrial programed stimulation or atrial programed stimulation following intravenous isoprenaline drip. In case 2, the A wave at the apex of the LAA occurred earliest during the operation. The catheter was perfused with cold saline, and the ablation parameters (43°, 20–30 W, 17 ml/min) were selected, with the pressure controlled at 5 g. Atrial tachycardia converted to sinus rhythm temporarily but returned to AT after a few seconds. Sinus rhythm and AT appeared alternately in the child after 40 s × 5 times consolidation ablation around this area. Because the best target was located at the apex of the LAA and the ablation risk was high, resection of the LAA was performed through the posterior lateral incision of the left chest combined with cardiothoracic surgery. In case 3, meticulous mapping was performed from outside to inside along the RAA. The earliest excitation point was marked at the tip of the middle part of the RAA, the ablation parameters (43°, 25–35 W, 17 ml/min) were selected, and the pressure was controlled around 5–10 g. Atrial tachycardia was terminated after acceleration, and consolidation ablation was performed for 60 s. After the operation, AT recurred, and thoracoscopic RAA resection was performed in combination with cardiothoracic surgery. During the atrial appendectomy, ECG monitoring suggested that atrial tachycardia was terminated after the atrial appendage was clamped. Figure 2 shows three-dimensional mapping shown in, and Figure 3 shows postoperative pathology.

**FIGURE 4:**
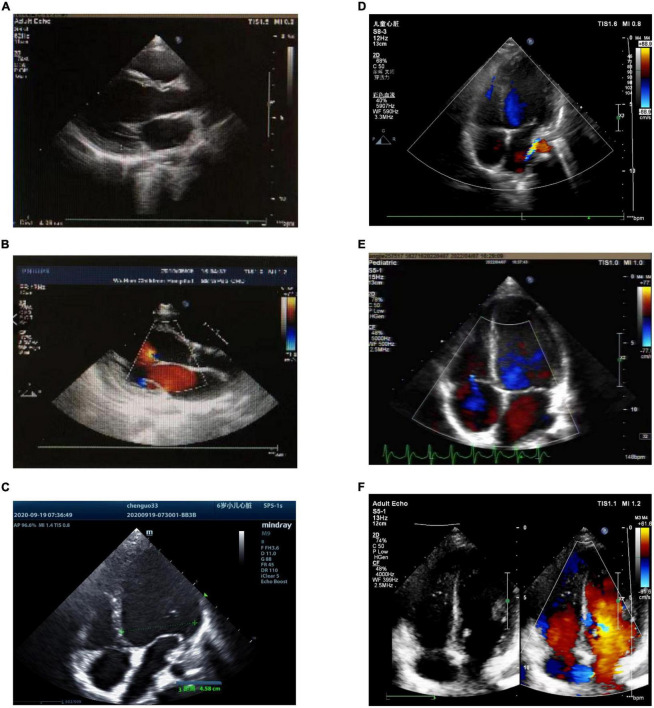
Preoperative and postoperative echocardiography of atrial tachycardia (AT) from the atrial appendage. **(A)** Case 1: preoperative echocardiography. Left ventricular end-diastolic diameter (LVEDD) 40 mm, left ventricular ejection fraction (LVEF) = 42%. **(B)** Case 1: echocardiography 1°month after radiofrequency catheter ablation (RFCA). LVEDD 37 mm, LVEF = 53%. **(C)** Case 2: preoperative echocardiography. LVEDD 45.8 mm, LVEF = 29%. **(D)** Case 2: echocardiography 1°month after LAA resection. LVEDD 37 mm, LVEF = 46%. **(E)** Case 3: the left picture shows preoperative echocardiography. LVEDD 45 mm, LVEF = 45%. **(F)** Case 3: echocardiography 1°month after RAA resection. LVEDD 42 mm, LVEF = 56%. LVEDD, left ventricular end-diastolic diameter; LVEF, left ventricular ejection fraction; RFCA, radiofrequency catheter ablation; LAA, left atrial appendage; RAA, right atrial appendage.

After the operation, all three children recovered sinus rhythm (Figure 1). Follow-up from 1°month to 1.5 years showed no recurrence of AT, and heart size and ejection fraction gradually recovered (Figures 1, 4 and Table 1).

**FIGURE 5:**
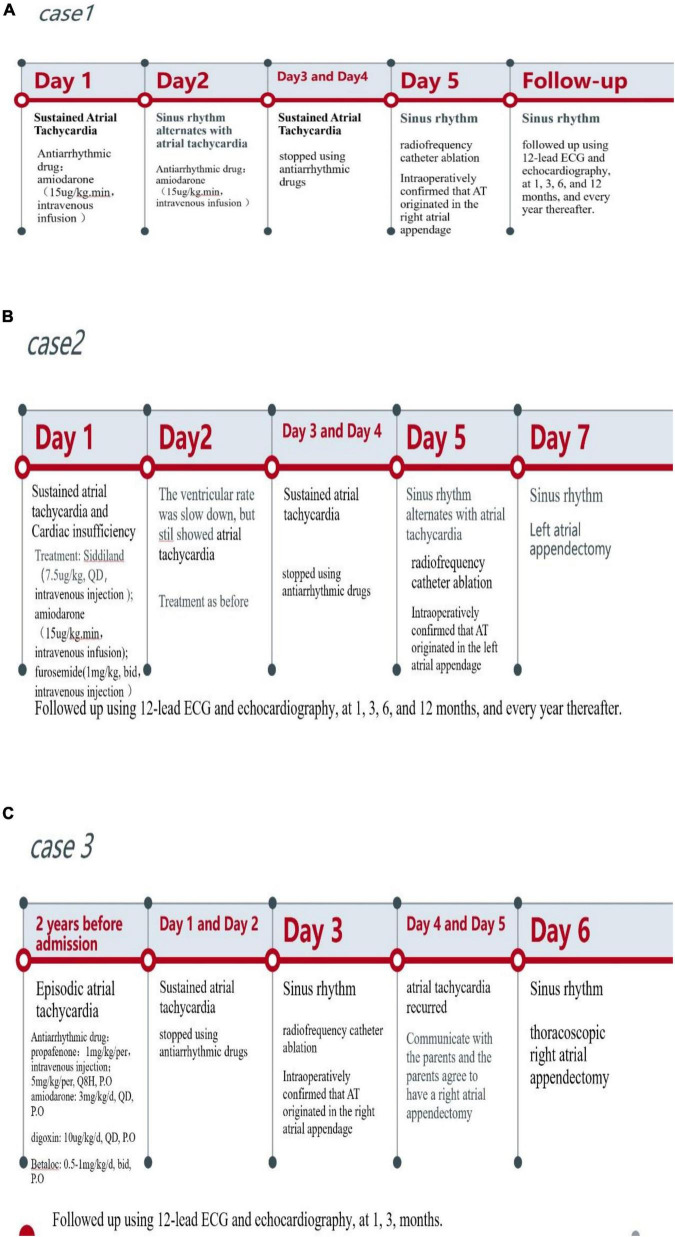
The timeline of the three cases. **(A)** The timeline of case 1. **(B)** The timeline of case 2. **(C)** the timeline of case 3.

The time line of the three cases showed in Figure 5.

## Discussion

Atrial tachycardia originating from the AA shows characteristic P wave changes on ECG. Electrocardiograms of AT originating from the RAA showed the following: (1) P waves in lead V1 were negative, some of which included notches, and the other precordial leads gradually move in a positive direction; (2) P waves in leads II, III, and aVF were positive; and (3) P waves in lead I were positive or on the equipotential line, were positive or negative in lead aVL, and were negative or on the equipotential line in lead aVR (11–13). In contrast, the ECG of AT from LAA showed the following: (1) P waves in lead V1 are positive; (2) P waves in leads II, III, and aVF were positive; and (3) P waves in leads I and aVL were negative (11, 14, 15). In this report, the P waves in the ECGs of 2 cases of AT from the RAA and 1 case of AT from the LAA were consistent with these characteristics.

The comb muscles on the inner wall of the AA are developed and have differing thicknesses. Atrial tachycardia originating from this site may have multiple excitation loci and may radiate in all directions, resulting in a low success rate of RFCA (8). Due to the thin myocardial tissue between the pectinate muscles of the AA, RFCA in this area can cause the perforation of the AA, resulting in pericardial tamponade. Therefore, AT originating from the AA is easily mapped and difficult to ablate, resulting in a high rate of recurrence (16). In the three cases in our study, we used the Ensite three-dimensional mapping system to guide mapping and ablation. Ablations were performed *via* a cold saline-infused catheter, and the pressure and ablation temperature were controlled. A contrast agent was introduced through the catheter to allow for better mapping of the earliest excitation point (17). Catheter introduction was carefully performed to reduce the risk of pericardial perforation and AA thrombosis. Successful ablation occurred immediately in one patient, one case could not be successfully ablated, and one case was successfully ablated, but AT recurred. In the last two cases, small incision LAA resection and thoracoscopic RAA resection were performed during cardiothoracic surgery, respectively. For failed ablation or postoperative recurrence of AT, AA resection resulted in less trauma, longer-lasting curative effects, and a better long-term prognosis (11, 18).

Compared with traditional transthoracic incision surgery, video-assisted thoracoscopic surgery results in less bleeding during the operation, faster recovery, and less scarring. However, only one case of video-assisted thoracoscopic surgery has been reported in a child (19). In addition, there was a report of a 13-year-old boy who underwent thoracoscopic clamp RFCA to treat AT that originated from the RAA following the failure of catheter ablation (20). Losantos reported a 21-year-old woman with LAA tachycardia who underwent successful ablation in the epicardium following two failed endocardial ablation procedures (21). Combined ablation and exclusion of the LAA were reported in a 10-year-old boy for treatment of AT using video-assisted thoracoscopic surgery (22). These cases provided valuable information regarding the treatment of AT originating from the AA.

Histopathological examination found that there were local degenerative and necrotic foci in the excised atrial appendage specimens, which were considered the ablation target. The myocardial cells showed granular degeneration, vacuolar degeneration, fibrous tissue proliferation, and inflammatory cell infiltration.

In our three cases, AT lasted from 3 days to 2 years, and each case resulted in TIC, which was characterized by increased LVEDD and decreased LVEF. Each of the three patients was treated with several antiarrhythmic drugs before undergoing RFCA, but no therapeutic effects were observed. After RFCA and AA resection, the ECGs showed recovery of sinus rhythm, and LVEDD and LVEF gradually recovered in each patient. These findings showed that cardiomyopathy caused by tachycardia was reversible. Studies have shown that the median duration required for LVEF to return to normal in children with TIC caused by AT was 1.5 months, while reverse ventricular remodeling required several months to years to complete (23, 24). In this report, two cases returned to normal LVEDD and LVEF at 1-year follow-up, and one case improved on the second day after surgery. However, long-term data were not available due to the short follow-up period. Additional follow-up appointments will be scheduled to monitor these patients.

In summary, AT originating from AA in children showed characteristic P-wave manifestations on ECG, and persistent AT resulted in TIC. For children who cannot be cured using RFCA or who relapse after an operation, AA resection may be a reliable therapeutic approach.

## Data availability statement

The original contributions presented in this study are included in the article/Supplementary material, further inquiries can be directed to the corresponding author.

## Ethics statement

The studies involving human participants were reviewed and approved by Wuhan Children’s Hospital. Written informed consent to participate in this study was provided by the participants’ legal guardian/next of kin.

## Author contributions

JL and XC proposed case ideas and participated in discussions. XF and DS assisted in collecting and summarizing cases. JL and CL wrote the original manuscript. YZ critically reviewed and proofread the manuscript. All authors contributed to the article and approved the submitted version.
