# Supplementary material for: Case report: Efficacy analysis of radiofrequency catheter ablation combined with atrial appendage resection for atrial tachycardia originating from the atrial appendage in children
Source: Front Cardiovasc Med. 2022 Oct 18;9:990325. doi: 10.3389/fcvm.2022.990325 (PMC9622751; doi:10.3389/fcvm.2022.990325)
Supplement: Supplementary file 1 [file Data_Sheet_1.ZIP › Data Sheet 1/Table 1.docx]

**Table 1: Clinical features and efficacy analysis of 3 cases of atrial tachycardia originating from the atrial appendage**

|  | | Case 1 | Case 2 | Case 3 |
| --- | --- | --- | --- | --- |
| Gender | | female | female | male |
| Age | | 3.5 years | 5.75 years | 12.9 years |
| Body mass(kg) | | 15 | 23 | 34 |
| Chief complaint | | Tachycardia was found for 5 days | Syncope once 3 days ago | Palpitation for 2 years |
| AT frequency (bpm) | | 200 | 160 | 145 |
| CK-MB (U/L) | | 26 | 17 | 17 |
| Troponin T (ng/ml) | | 0.068 | 0.113 | 0.066 |
| BNP (pg/ml) | | >9000 | 2768 | 181 |
| Preoperative ECG | | Sustained AT | Sustained AT | Sustained AT |
| The orgin of AT | | RAA | LAA | RAA |
| Preoperative LVEDD (mm) | | 40 | 45.8 | 45 |
| Preoperative LVEF (%) | | 42 | 29 | 45 |
| antiarrhythmic agents | | amiodarone | cedilanid, amiodarone | propafenone, amiodarone, digoxin, betaloc |
| treatment | | RFCA | RFCA combined with LAA resection | RFCA combined with thoracoscopic RAA resection |
| Postoperation of RFCA | ECG | sinus rhythm | paroxysmal AT | AT |
|  | LVEDD (mm) | 39 | - | - |
|  | LVEF (%) | 50 | - | - |
| Postoperation of AA resection | ECG | - | sinus rhythm | sinus rhythm |
|  | LVEDD (mm) |  | 42.6 | 44 |
|  | LVEF (%) |  | 33 | 52 |
| 1 month after the operation | ECG | sinus rhythm | sinus rhythm | sinus rhythm |
|  | LVEDD (mm) | 36 | 37 | 42 |
|  | LVEF (%) | 53 | 46 | 56 |
| 1 year after the operation | ECG | sinus rhythm | sinus rhythm | - |
|  | LVEDD (mm) | 35 | 35 |  |
|  | LVEF (%) | 65 | 60 |  |

bpm, beats per minute; CK-MB, creatine kinase-MB; BNP, brain natriuretic peptide; ECG, electrocardiogram; AT, atrial tachycardia; RAA, right atrial appendage; LAA, left atrial appendage; LVEDD, left ventricular end diastolic diameter; LVEF, left ventricular ejection fraction; RFCA, radiofrequency catheter ablation; AA, atrial appendage.
