# Supplementary figures and images for: Case report: Efficacy analysis of radiofrequency catheter ablation combined with atrial appendage resection for atrial tachycardia originating from the atrial appendage in children
Source: Front Cardiovasc Med. 2022 Oct 18;9:990325. doi: 10.3389/fcvm.2022.990325 (PMC9622751; doi:10.3389/fcvm.2022.990325)

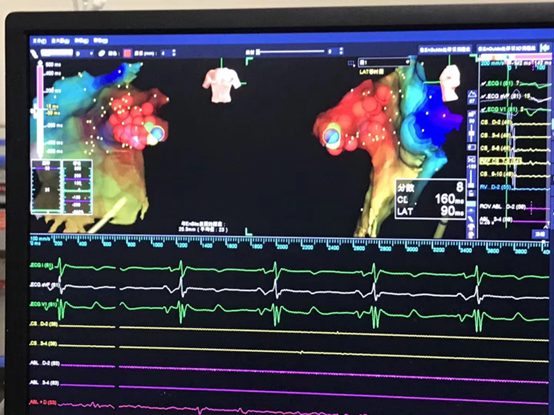

Supplement: Supplementary file 1 [file Data_Sheet_1.ZIP › Data Sheet 1/Figure2 (A).tif]

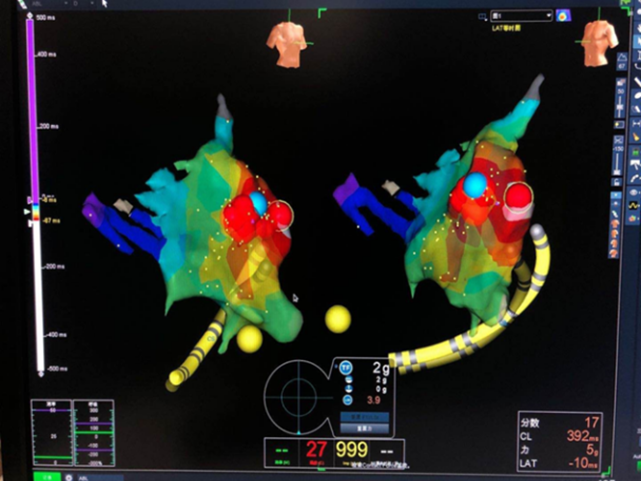

Supplement: Supplementary file 1 [file Data_Sheet_1.ZIP › Data Sheet 1/Figure2ú¿B).tif]

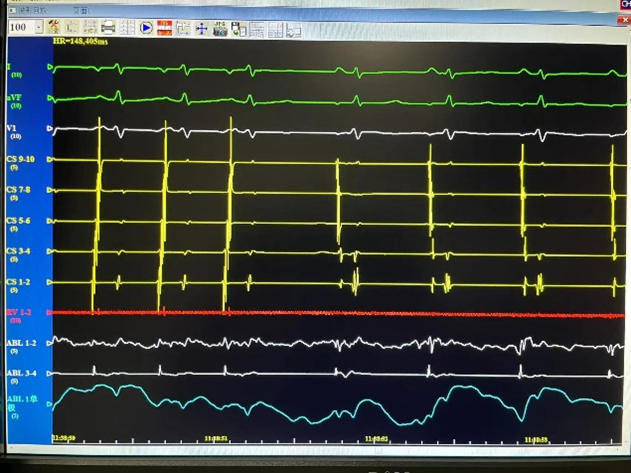

Supplement: Supplementary file 1 [file Data_Sheet_1.ZIP › Data Sheet 1/Figure2ú¿Cú⌐.tif]

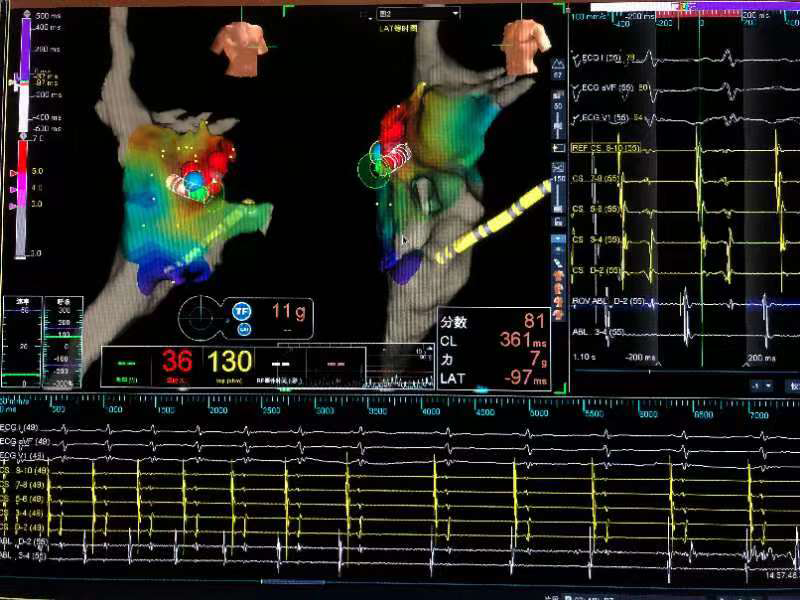

Supplement: Supplementary file 1 [file Data_Sheet_1.ZIP › Data Sheet 1/Figure2ú¿Dú⌐.tif]

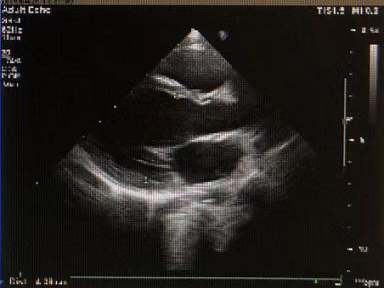

Supplement: Supplementary file 1 [file Data_Sheet_1.ZIP › Data Sheet 1/figure3(A).tif]

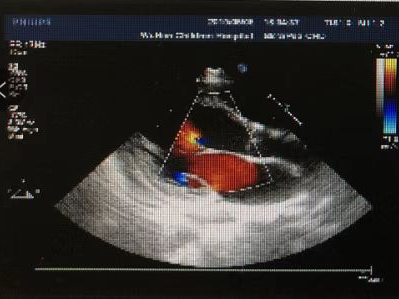

Supplement: Supplementary file 1 [file Data_Sheet_1.ZIP › Data Sheet 1/figure3(B).tif]

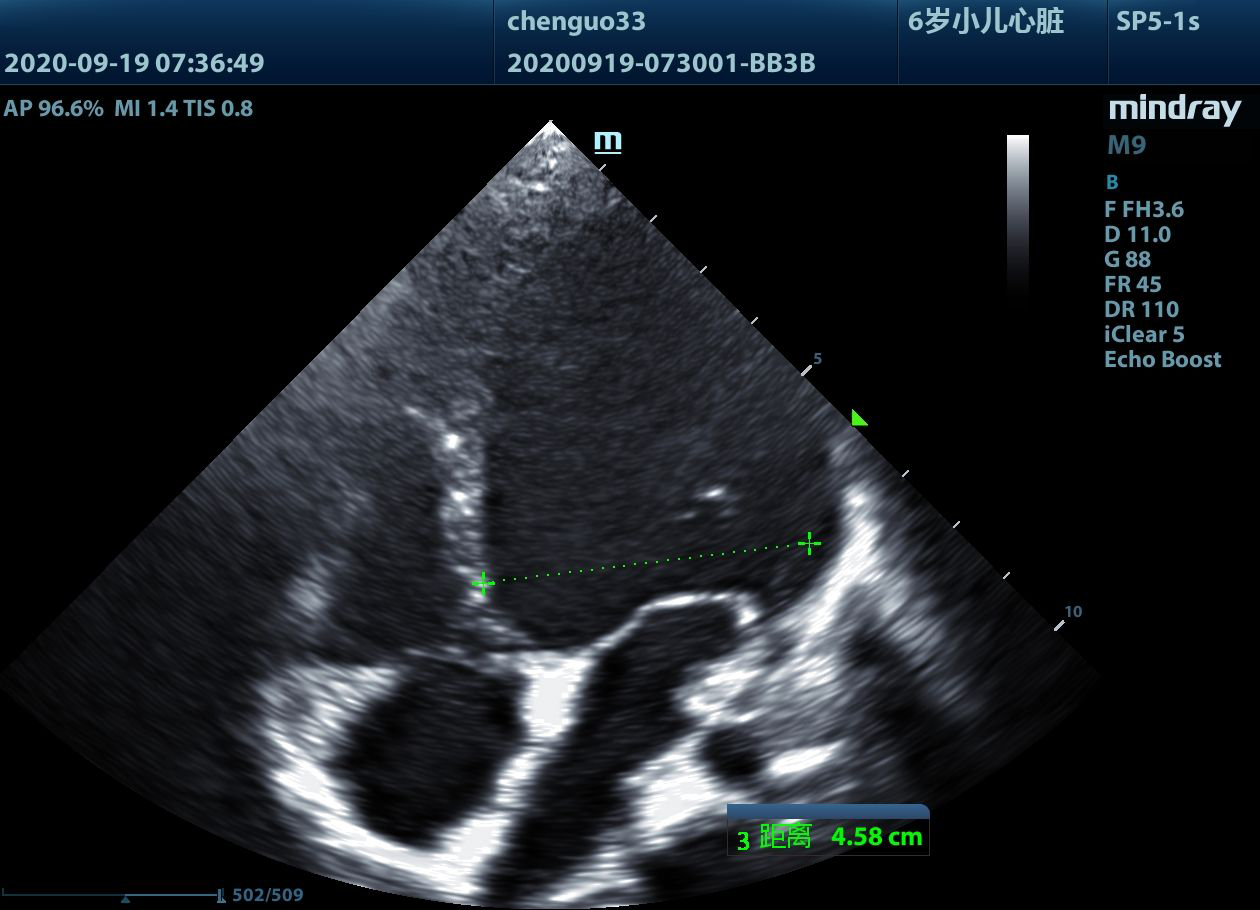

Supplement: Supplementary file 1 [file Data_Sheet_1.ZIP › Data Sheet 1/Figure3ú¿Cú⌐.tif]

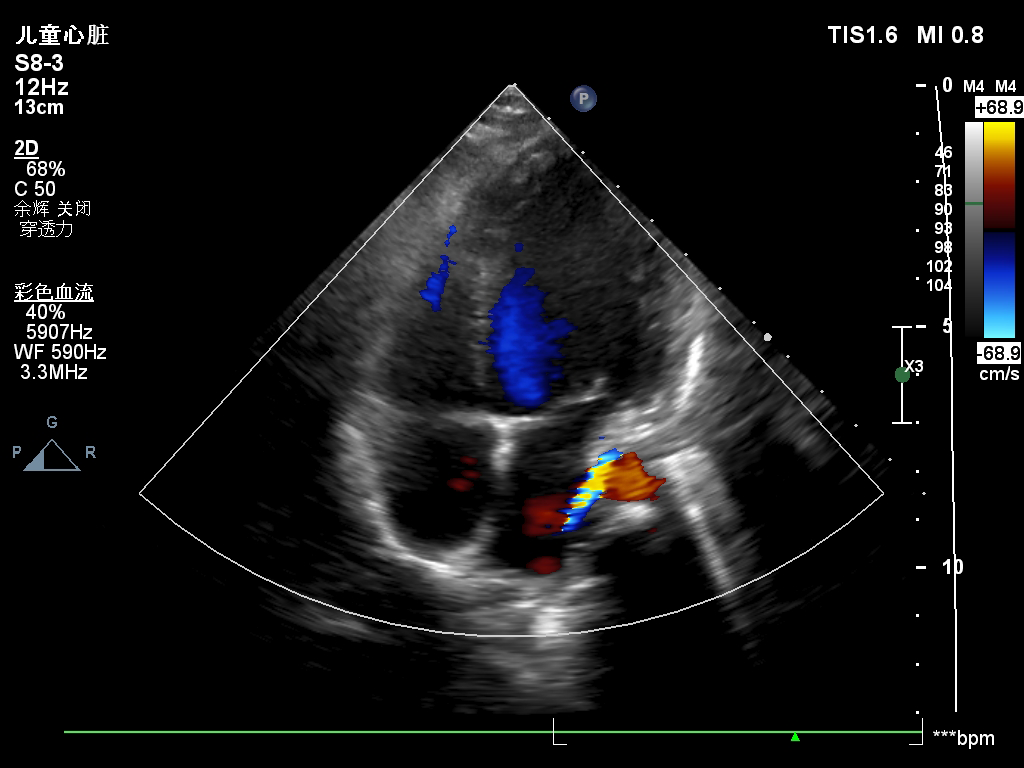

Supplement: Supplementary file 1 [file Data_Sheet_1.ZIP › Data Sheet 1/Figure3(D).tif]

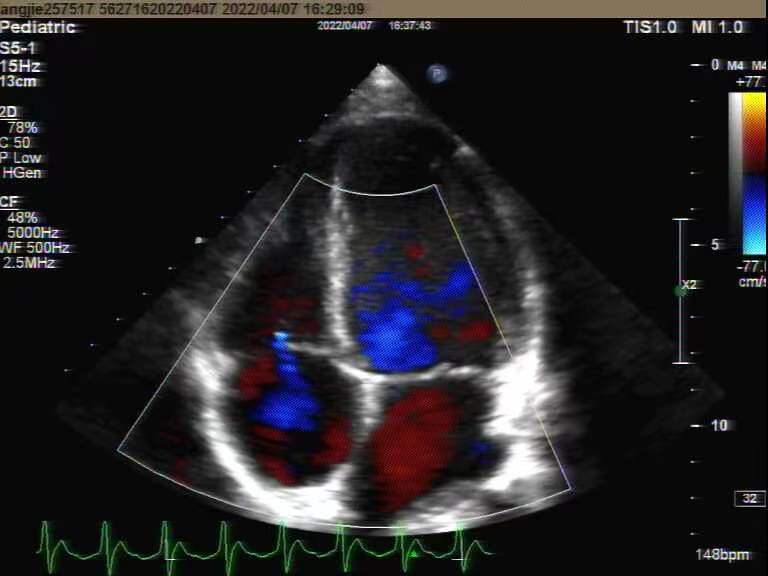

Supplement: Supplementary file 1 [file Data_Sheet_1.ZIP › Data Sheet 1/figure3(E).tif]

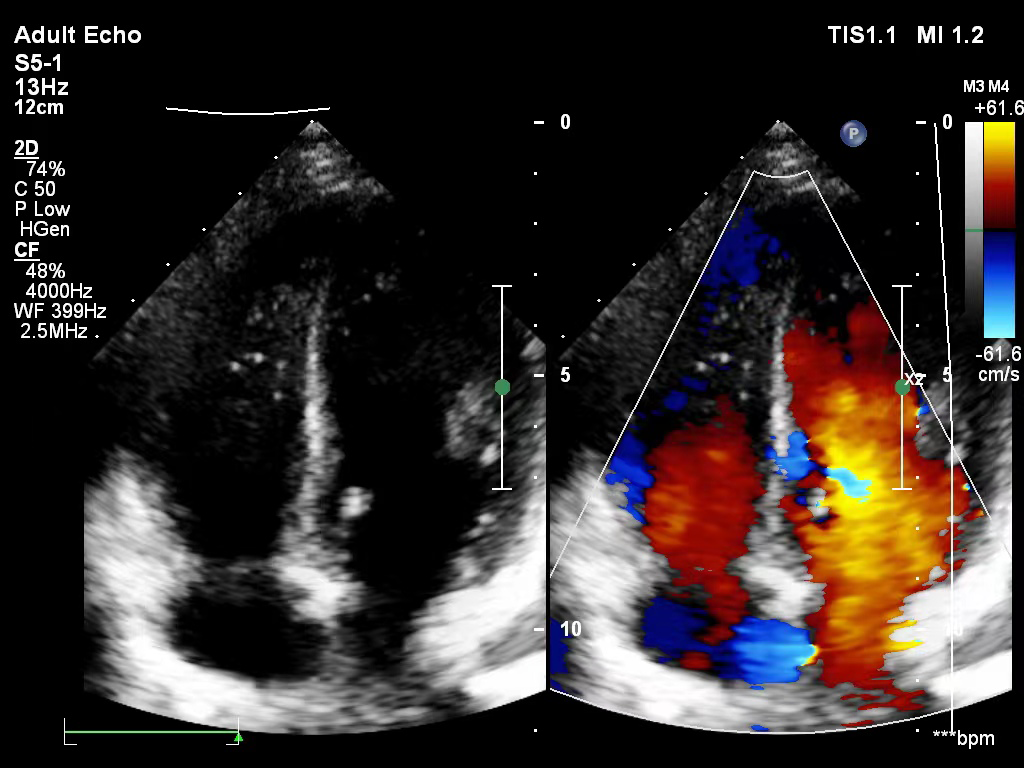

Supplement: Supplementary file 1 [file Data_Sheet_1.ZIP › Data Sheet 1/figure3(F).tif]

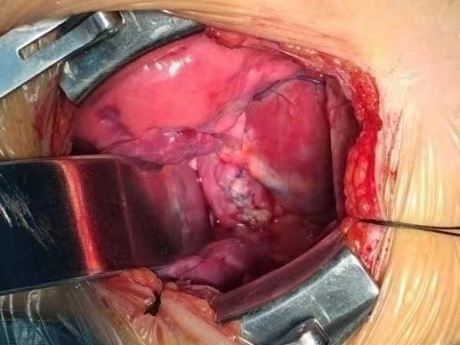

Supplement: Supplementary file 1 [file Data_Sheet_1.ZIP › Data Sheet 1/Figure4(A).tif]

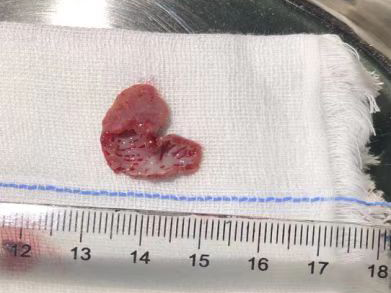

Supplement: Supplementary file 1 [file Data_Sheet_1.ZIP › Data Sheet 1/Figure4(B).tif]

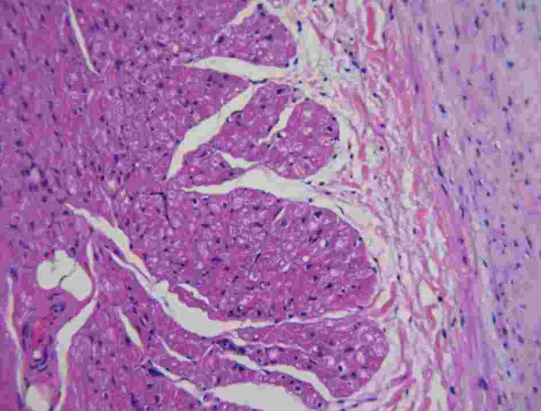

Supplement: Supplementary file 1 [file Data_Sheet_1.ZIP › Data Sheet 1/Figure4(C).tif]

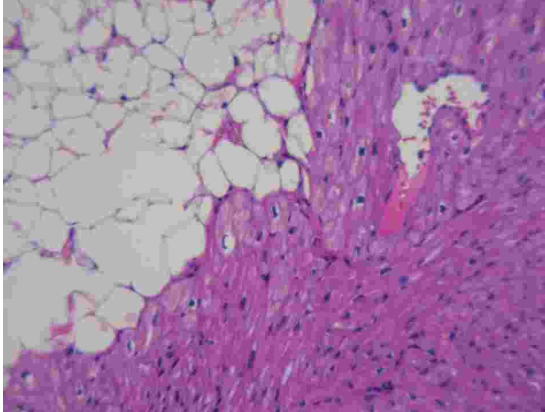

Supplement: Supplementary file 1 [file Data_Sheet_1.ZIP › Data Sheet 1/Figure4(D).tif]

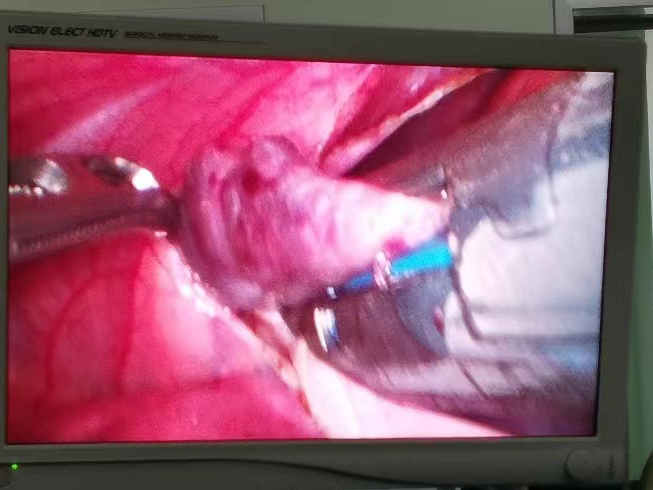

Supplement: Supplementary file 1 [file Data_Sheet_1.ZIP › Data Sheet 1/Figure4(E).tif]

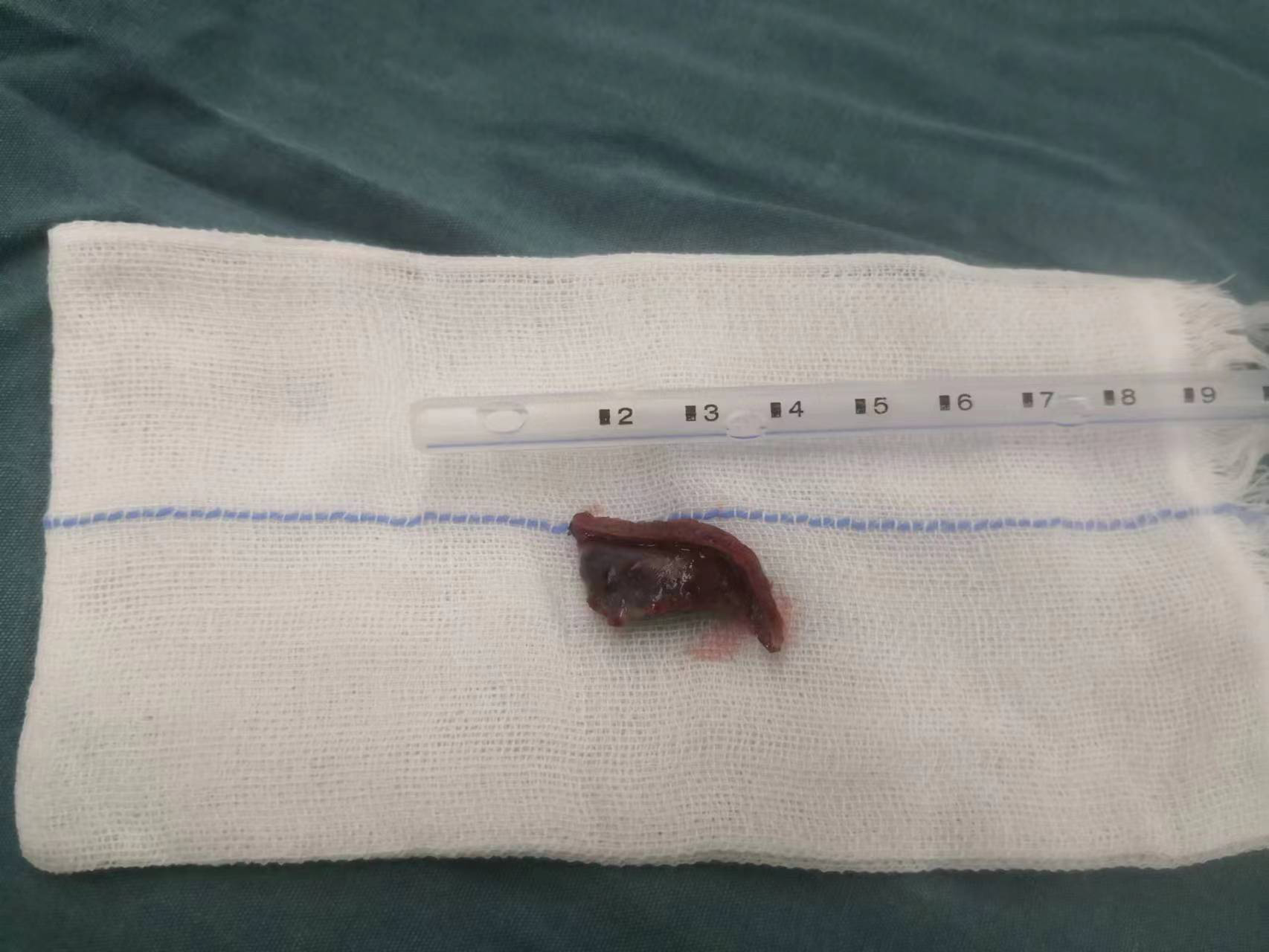

Supplement: Supplementary file 1 [file Data_Sheet_1.ZIP › Data Sheet 1/figure4(F).tif]

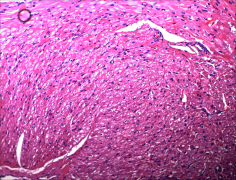

Supplement: Supplementary file 1 [file Data_Sheet_1.ZIP › Data Sheet 1/Figure4(G).tif]

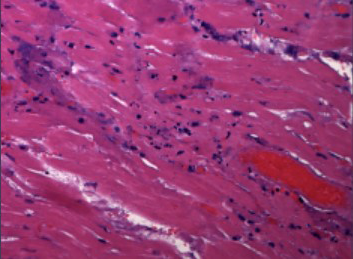

Supplement: Supplementary file 1 [file Data_Sheet_1.ZIP › Data Sheet 1/Figure4(H).tif]

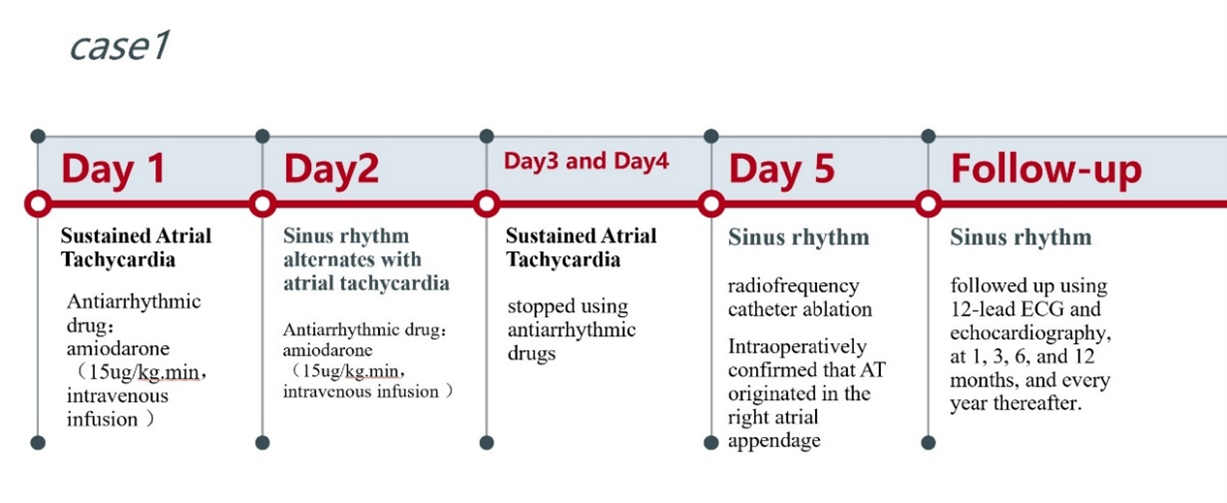

Supplement: Supplementary file 1 [file Data_Sheet_1.ZIP › Data Sheet 1/Figure5ú¿Aú⌐.tif]

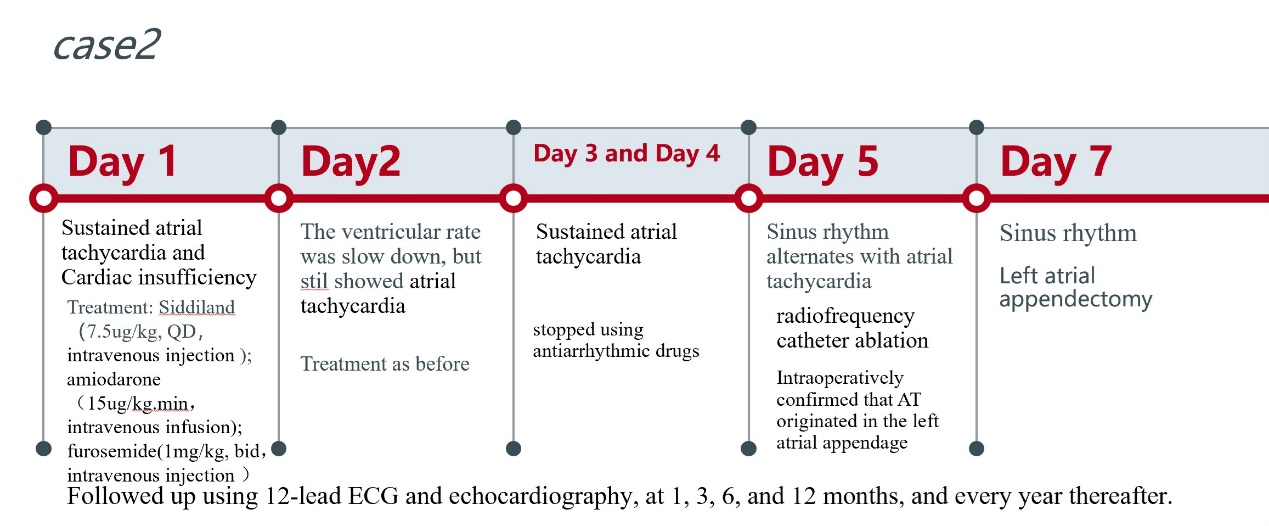

Supplement: Supplementary file 1 [file Data_Sheet_1.ZIP › Data Sheet 1/Figure5ú¿Bú⌐.tif]

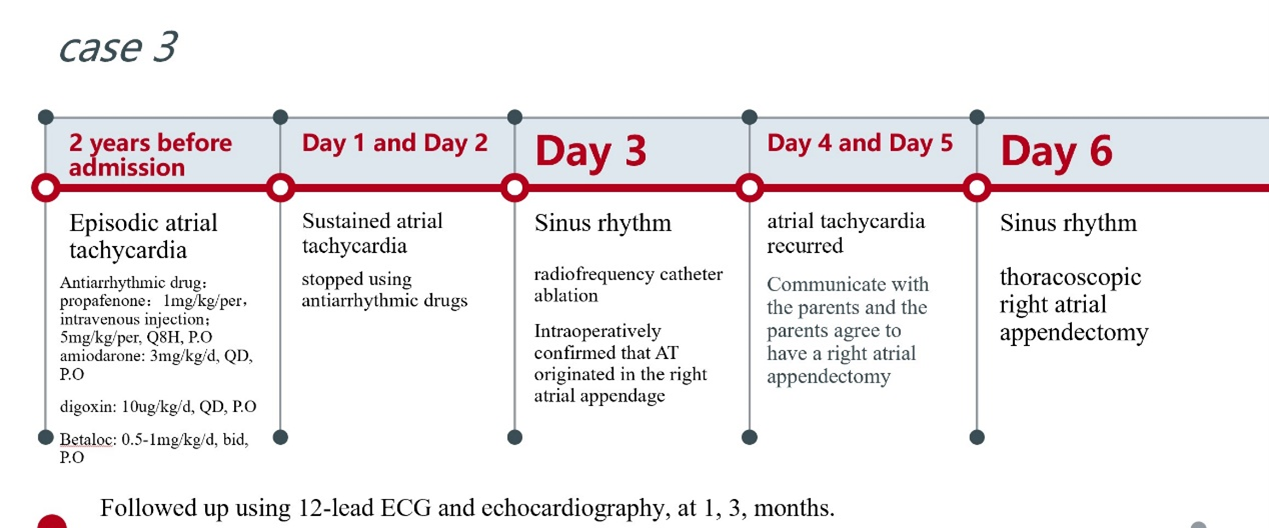

Supplement: Supplementary file 1 [file Data_Sheet_1.ZIP › Data Sheet 1/Figure5ú¿Cú⌐.tif]
